# From Manual to Automated: Exploring the Evolution of Switchover Methods in Injection Molding Processes—A Review

**DOI:** 10.3390/polym17081096

**Published:** 2025-04-18

**Authors:** Christian Bielenberg, Markus Stommel, Peter Karlinger

**Affiliations:** 1 Department of Plastics Technology, Faculty of Engineering Sciences, Rosenheim Technical University of Applied Sciences, Hochschulstraße 1, 83024 Rosenheim, Germany; 2 Faculty of Mechanical Science and Engineering, TUD Dresden University of Technology, Helmholtzstr. 10, 01062 Dresden, Germany; 3 Institute Polymer Materials, Leibniz-Institute for Polymer Research Dresden, Hohe Straße 6, 01127 Dresden, Germany

**Keywords:** plastic injection molding, switchover, switchover, v/p, adaptive control, machine learning, process control

## Abstract

Thermoplastic injection molding is a widely used process for producing complex three-dimensional plastic parts with tight dimensional tolerances. A key determinant of part quality is the switchover point—the transition from velocity-controlled filling to pressure-controlled packing. This transition affects critical product attributes, such as d imensional accuracy, weight consistency, and surface finish. Precise control of the switchover point enhances process stability, robustness, and adaptability. This review consolidates recent advancements in switchover methods and adaptive control techniques. Improvements in traditional methods include the use of pressure gradient detection to mitigate viscosity variations and adaptive control to refine stroke- and time-dependent switchovers. In addition, deformation-based strategies detect the mold-opening force associated with cavity pressure through clamping force, mold separation, or tie-bar elongation. The integration of machine learning and feature extraction techniques enables the real-time adjustment of the switchover point by mapping relationships between process parameters and quality criteria. In addition, ultrasonic sensors provide non-invasive melt front detection, reducing the risk of mold damage. Real-time simulations, updated through nozzle pressure feedback, complement these methods to achieve precise switchover timing. This review also identifies persistent challenges, such as sensitivity to material properties, machine wear, and environmental conditions, and it explores future directions for improving the accuracy and adaptability of switchover control in modern injection molding processes.

## 1. Introduction

**Figure 1:**
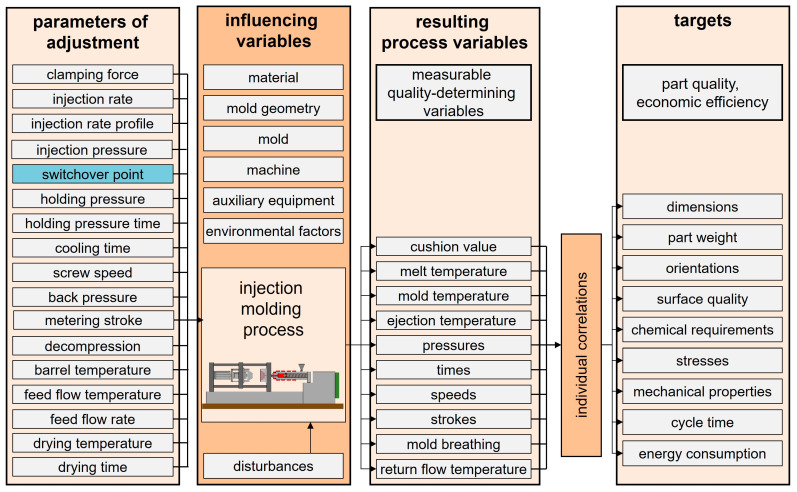
Representative parameters and variables for the injection molding process.

Thermoplastic injection molding is employed to manufacture complex three-dimensional plastic components that are subject to precise tolerances. The injection molding process consists of the stages of mold closing and clamping, filling, packing and holding, cooling and metering, and mold opening and ejecting. Those phases may overlap to some degree and are adjusted through the adjustable process variables. The quality and uniformity of molded products depends largely on the ability of the injection molding machine to fill the cavity completely and to compensate for the volumetric shrinkage of the plastic during cooling by maintaining adequate pressure. The switchover point in injection molding, which refers to the transition from velocity-controlled filling to pressure-controlled packing, plays a decisive role in determining part quality [1,2]. This transition directly affects key part attributes such as dimensional accuracy [3], weight consistency [4], and surface finish [5]. The switchover point is one of several adjustable process parameters. In interactions with the boundary conditions, these parameters result in characteristic process variables. These variables determine part quality. A representation of such parameters, variables, and resulting quality indicators is shown in Figure 1.

The ability to precisely control the injection molding process has become an essential factor in improving both product quality and process stability [6]. Advancements in data collection and processing, in conjunction with sensing options such as cavity sensors [3,7], nozzle sensors [8,9,10,11,12], ultrasonic sensors [13,14,15], and tie-bar strain sensors [1,16,17], are allowing for new approaches for process control.

The present review is centered on the switchover point and is intended to analyze and consolidate the state of the art and scientific advances related to the control and optimization of the switchover point in injection molding. By exploring the technological developments over the past decades, this paper aims to highlight how these innovations have helped to improve process reliability, efficiency, and part consistency. This review also seeks to identify the current challenges associated with switchover control, such as the sensitivity of the process to material properties, machine wear, and environmental factors.

To provide a comprehensive understanding, this paper first outlines the basic principles governing the switchover point, followed by an examination of the common challenges manufacturers face in managing this critical phase. Those challenges include process adaptations, the closing behavior of the non-return valve (NRV), in-mold sensor positioning and response time, as well as multi-cavities. In addition to reviewing technologies that lower the impact of influencing factors, such as the closing behavior of the NRV, and therefore contribute to more robust switchover control, the focus will be on the technological innovations that directly impact the switchover point itself and the adaptive control of the switchover point. This review presents methodologies for the management of switchovers for multi-cavities, as well as approaches that utilize changes in the pressure gradient as opposed to traditional pressure thresholds. Additionally, an examination of adaptive methodologies is conducted, encompassing the control of the reference point for the switchover or the switchover point through pressure integrals; mold separation, and tie-bar elongation; adapted filling simulation; and the utilization of artificial intelligence.

Lastly, this review aims to provide a detailed account of how these advances have been integrated into modern injection molding processes and to offer insight into future directions for improving the accuracy and adaptability of switchover control.

## 2. Definition and Significance of the Switchover Point

The switchover point from the filling to holding phase should occur when volumetric filling is reached. During volumetric filling, the molded part is filled but not yet molded. At this transition, the control variable changes from the injection rate to pressure [18].

**An early switchover** will result in a premature stopping of the melt front and a filling at holding pressure. This may result in flow marks, incomplete filling, higher shrinkage potential, sink marks, poor weld lines, as well as weight variations [19,20].

**A late switchover** results in high-pressure peaks, increased mold clamping force requirements, as well as an unnecessarily high compaction, which can lead to over-injection (burrs and flashes), mold damage, internal orientation due to mold discharge, and higher internal stresses, making the molded parts more susceptible to breakage [19,20].

**A proper switchover point** is reached at the volumetric filling stage, with the maximal cavity pressure occurring subsequently as the melt is compressed [21]. In the case of mold geometries that have a high flow-length-to-wall thickness ratio, the switchover point should be selected based on the required part properties far from the gate to avoid insufficient compensation during the holding pressure phase [3]. However, even the optimal switchover point is only set during the setup phase and will vary as the production environment changes [5]. It is recommended that, where possible, a machine operator sets a switchover point that is slightly before the optimal point to avoid a late switchover [22].

**Figure 2:**
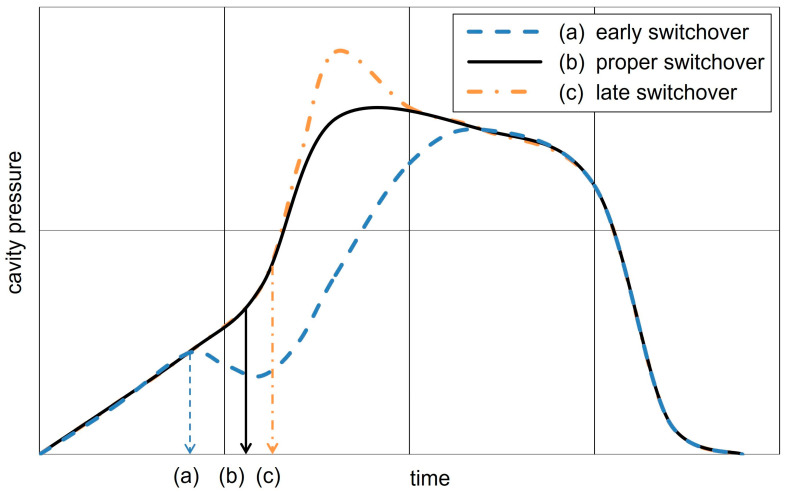
Schematic representation of the cavity pressure curve for a too early (a), proper (b), and too late (c) switchover point based on the concepts illustrated in Figure 2 of [17], Figure 1 of [23], and Figure 2 of [24].

As demonstrated in numerous publications, the resulting cavity pressure curve is indicative of a switchover that is either too early, too late, or executed in a proper manner. To provide a more compact overview, the cavity pressure plots from [17,23,24] have been combined into a single scheme, which is shown in Figure 2.

**Identifying the desired switchover point:** The standard procedure for setup is a filling study, in which the injection pressure for the holding pressure phase is reduced to zero, and the volumetric filling is determined by adjusting the switchover point [25]. It is not advisable to perform direct metering after an injection when an open nozzle makes contact with the cavity, as this can result in the ingress of molten material into the cavity. It is therefore recommended to introduce a delay to allow the gate to freeze [26].

It is important to maintain a sufficient melt cushion after the holding phase, to avoid process instability. If the melt cushion is insufficient, the process must be adjusted and the switchover point must be checked and adjusted as necessary [20]. Once the switchover point has been established, further process optimization takes place. Subsequently, the switchover point should be checked once more with the post-optimization parameters [25]. It is also worth noting that process settings and switchover points can differ between machines, even if they have identical capabilities [27,28].

### 2.1. Traditional Methods for Switchover Management

The following are the standard methods for switching from velocity-controlled filling to pressure-controlled holding [25]:

- Time-dependent switchover:

The velocity-controlled injection phase stops after a predefined time.

- Stroke-dependent switchover:

A switchover occurs when the screw reaches a specific position or stroke length.

- Pressure-dependent switchover:

A switchover occurs when a threshold injection pressure is reached.

- In-mold-sensor-dependent switchover (mostly cavity pressure):

A switchover occurs when the measured value reaches a predefined threshold.

As observed by Kazmer, stroke-dependent and pressure-dependent switchovers are reliable methods for most applications. The integration of in-mold sensors further enhances the reliability of this approach. The use of high-speed hardware and software is essential for external controls to meet the stringent response time requirements [29]. A stroke-dependent switchover is the most prevalent approach as it is more effective in handling variations in melt viscosity compared to pressure- or time-based methods [25].

In-mold sensors are preferred for their accuracy in detecting the melt front and their capacity to trigger a switchover at a consistent fill level. These in-mold sensors, which include pressure sensors and also a variety of other types such as optical and ultrasonic ones, contribute to process and quality consistency. However, their widespread use is limited by the invasive nature of the installation, the often considerable cost involved, and the impracticality of using them in certain mold geometries. Furthermore, they can result in visible marks on the surface of the molded part and increase the maintenance costs associated with the molding machines in which they are used [4,13,25,30,31,32].

### 2.2. Injection Molding Processes Without Switchover

It is possible to operate an injection molding machine without any switchover. One possible solution is to abandon the velocity-controlled filling method and instead set the injection pressure to match the holding pressure. However, especially with thin- or thick-walled parts, the fastest possible injection is desired, so injection pressures higher than the holding pressure may be required. The elevated pressure during the compression phase within the mold induces an increased mold-opening force, requiring the application of a greater clamping force. This may result in the use of a larger machine than is strictly necessary due to the higher clamping force required [25].

Still, there are applications for a process that does not necessitate a transition from velocity to pressure control. For example, the LEGO process employs adaptive control to guarantee uniform brick release, with the injection pressure calibrated to match the holding pressure. This eliminates the necessity for a defined injection rate or switchover point. The process relies on parameters such as the injection pressure, melt temperature, injection time, and flow rate, which are averaged over the last 10 cycles to maintain stability. However, this approach requires higher clamping forces, necessitating larger machines [25].

Furthermore, iMFLUX offers pressure-controlled injection without a switchover, which is particularly advantageous for stabilizing recycled material with viscosity fluctuations [33,34]. According to the findings of Krantz et al. [35], pressure-controlled filling resulted in a slight improvement in tensile strength compared to velocity-controlled filling. This effect was attributed to how the slower filling rate accompanies pressure-controlled filling, which has been associated with enhanced surface layer orientation. However, it is important to note that the slower injection rate of pressure-controlled filling may limit its effectiveness for thin-walled parts.

Engel Austria GmbH’s X-Melt process represents another method without a switchover, whereby the material expands into the cavity upon opening the nozzle following the buildup of pressure. This method is applicable to the production of thin-walled and micro-precision parts as it exploits the ability of the plastic melt to expand at high-preload pressures [36,37].

### 2.3. A Comparison of Switchover Methods

A time-dependent switchover is regarded as the least robust method [27,38], with a slight advantage over a speed-dependent switchover [39,40]. (Aloise et al. introduced an automatic speed-dependent switchover, using threshold values for deceleration during the transition to holding pressure [31]. Offergeld further refined this approach by employing optimization strategies to minimize screw acceleration during this phase [41].) A stroke-dependent switchover offers enhanced robustness; however, it can be influenced by nozzle leaks or alterations in the material. Hydraulic pressure, when utilized as an indicator for a pressure-dependent switchover, is deemed less reliable due to potential delays in melt compressibility. Consequently, nozzle pressure is favored due to the reduced plastic mass between the cavity and the sensor. The most preferred method for a pressure-dependent switchover is cavity pressure, as it provides direct information from within the mold [38].

In-mold sensors at the end of the flow are regarded as the most robust switchover technique, particularly in the context of handling viscosity changes [27,38,39,40,42]. Schubert et al. [42] have shown that pressure-dependent switching via cavity sensors can cause backflow and fluctuations, particularly with more compressible materials. These effects can be mitigated by using in-mold sensors at the end of the flow path. Diao et al. [32] compare ultrasonic sensors with the stroke position and post-gate cavity pressure sensors. They found that although the ultrasonic sensor does not require any cavity rework, it only provides a signal that can be used for switching when the melt front is reached. Consequently, the position of the ultrasonic sensor must be varied and determined according to the desired switchover point.

Schubert et al. [42] and Johnson et al. [43,44] compared the robustness of a stroke-dependent switchover with various in-mold-sensor-based switchover techniques for different injection molding processes featuring single or multiple injection rates. These comparisons also considered different viscosities [43,44], different materials, and wall thicknesses [42]. 

The comparisons reviewed indicate that no particular switchover method consistently outperforms the others for all possible combinations. The selection of the ideal switchover method, as well as the choice of the most suitable in-mold sensor type and position, are mainly dependent on the injection process [42,43,44], expected viscosity changes [43,44], rheological properties of the material [45], quality criteria [27], and wall thickness [42].

## 3. Challenges and Limitations Associated with the Switchover Point

The optimal switchover point varies due to the influence of process adaptations, as well as variations in the environment and material [40,42]. These variations may influence the melt viscosity and thus directly affect the closing behavior of the NRV [46]. Additional challenges associated with in-mold sensors and multi-cavities are presented below. This section reviews these challenges and the limitations they impose on achieving a robust and reliable switchover point.

**Process adaptations:** As demonstrated by Ghose et al., several factors, including the injection rate, mold preheating, and nozzle temperature, directly influence the filling process [47]. When machine parameters such as the injection rate or temperature are varied, it is also necessary to calibrate alternative switchover methods that are less dependent on the filling behavior, such as a pressure-dependent switchover, in order to avoid potential errors [21,48].

**Figure 3:**
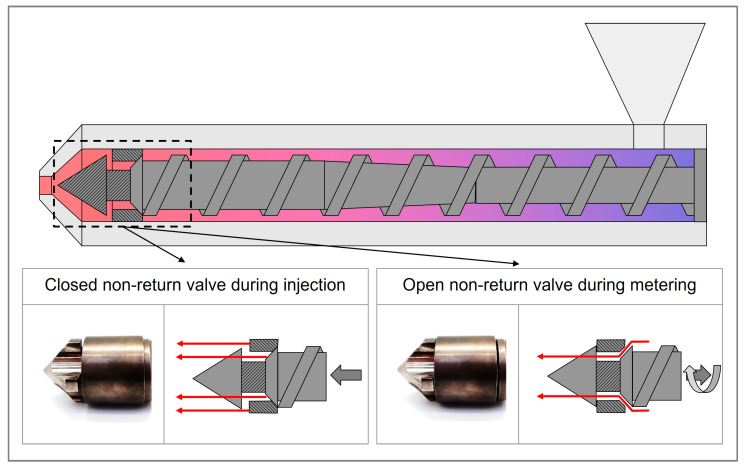
Schematic of a non-return valve in open positions during metering and closed positions during injection. Red arrows indicate the flow path for the polymer melt, while gray arrows represent the associated screw movement of the corresponding phases.

**Closing behavior of the non-return valve (NRV):** The NRV is designed to provide the unidirectional flow of the molten polymer. It remains open during metering to allow material accumulation and closes during injection to prevent backflow from the screw antechamber into the screw channel. Figure 3 illustrates the valve’s position in both open and closed states, corresponding to the metering and injection phases of the process. 

The way in which the NRV closes has a significant effect on the shot volume prior to the switchover point. If the valve closes prematurely, the resulting compression of the melt can result in an increase in the injection pressure. Conversely, if the valve closes at a late point in the cycle, the melt may flow back from the screw antechamber towards the screw, which can result in a drop in the pressure. Fluctuations in viscosity, for example resulting from temperature variations or alterations in material properties, can precipitate a premature or delayed closure of the valve, thereby introducing further complications to the process. Materials with lower viscosity lead to a faster closing of the NRV, while materials with higher viscosity favor a delayed closing of the NRV [45,49]. During operational observation, fluctuations in the injection pressure of approximately ±30 bar have been documented, with some of these fluctuations attributed to the inconsistent behavior of the valve [25].

**In-mold sensor positioning and response time:** The type of sensor and its positioning have a considerable influence on the effectiveness of a switchover using in-mold sensors. To detect volumetric filling, sensors at the end of the flow path are desirable. However, a position as close to the sprue as possible is generally recommended, as this is the only way to map the entire process sequence of the filling and holding phase. It is also important to consider the response time and information requirements. For instance, IR sensors can react more rapidly than contact temperature measurements. However, it is necessary to calibrate them according to the emissivity of the plastic melt that is to be processed [50]. In comparison, cavity pressure sensors have a lower response time, and Baesso et al. addressed this by developing a method for faster cavity pressure detection [51]. Ageyeva et al. [7] provide a comprehensive review of in-mold process monitoring, emphasizing the expanding role of sensors in Industry 4.0 applications. 

**Multi-cavities:** A cavity-pressure-based switchover is often less effective in these cases due to uneven filling. Therefore, a stroke-dependent switchover is a preferred option [52]. Ensuring uniform filling in multi-cavity molds represents a substantial challenge. In their study, Cooney et al. [52] used a naturally balanced runner system, which, in comparison to rheologically balanced systems, has the capacity to achieve an even filling, even in the event of process alterations such as an adjusted injection rate. Kazmer and Barkan [53,54] describe a system comprising multiple cavity pressure sensors and valves in the runner system which enables dynamic adjustment for the uniform filling of multiple cavities or cavities with multiple sprues. Ortgies [55] describes an application where the flows in the cavities of a multi-cavity mold are equipped with temperature sensors. This allows the hot runner to be automatically balanced according to the filling times of the cavities. The balanced hot runner can then be used for a switchover by reaching a representative temperature sensor.

## 4. Technological Advances for the Switchover Point

Advances in process monitoring and control have led to a variety of strategies to improve the switchover methods in injection molding. A systematic categorization of the reviewed contributions reveals two overarching groups, which are examined in detail in the following subsections:

- Lowering the impact of the influencing factors by decreasing disturbances or enhancing the process’s robustness to them. This includes strategies focused on a smooth transition between the filling and holding phase, incorporating NRV behavior, refining the switchover methods for multi-cavities, and rethinking pressure-dependent-switchover logic.
- Adaptive process control approaches leverage sensor data, simulations, or machine learning techniques to dynamically adjust the switchover point. This cluster includes step-by-step optimization methods, the use of pressure integrals, deformation-based approaches (such as mold separation and tie-bar elongation), filling simulation models, and artificial intelligence.

Despite addressing different aspects, the two clusters exhibit significant conceptual and methodological overlap. Emerging solutions bridge multiple domains, as exemplified by approaches for adaptive process control. These approaches use pressure integrals to maintain a constant shot volume, while simultaneously adjusting the pressure integral in accordance with the closure of the NRV [46,56].

### 4.1. Lowering the Impact of the Influencing Factors

At the switchover point, the control variable changes from injection rate to pressure, requiring a shift between two separate controllers [18]. This transition introduces a vulnerability to disturbances. This section reviews strategies aimed at reducing such sensitivities by either minimizing their root causes or enhancing the process’s robustness. Key approaches include improving the smoothness of the phase transition, compensating for dynamics associated with the NRV, refining pressure-based-switchover logic, and adapting the switchover methods for multi-cavities.

**Focusing on a smoot transition:** One common approach is to decrease the injection rate prior to the switchover point, enabling a bump-less transition between the filling and holding phases [57,58]. To do so and to enable a more seamless transition between the filling and holding pressure phases, Zheng et al. introduced a model that employs two iterative learning controllers. These are connected by a smoother transition scheme to control electro-hydraulic injection molding machines, thus enhancing transition efficiency [18]. Similarly, Froehlich et al. developed model predictive control for servo-pump-driven machines which improves process control, especially when executing defined injection or pressure profiles without knowledge of mold geometry or previous cycles [59,60]. In addition to these developments, Hopmann and Hornberg identified a deficiency in the transition between the two control units for velocity and pressure. They addressed this problem by using only one control unit and controlling cavity pressure solely through the injection rate, resulting in a more consistent cavity pressure profile in line with the reference trajectory [61]. Stemmler et al. and Hornberg et al. continued this line of research by developing cross-phase control systems that eliminate the need for separate control circuits during filling and holding. These systems use a mathematical model of the servo-electric drive, plasticizing unit, and cavity dynamics, which allows for smoother transitions [62,63]. Hornberg et al. applied this method to post-consumer recyclates, addressing the challenge of unavailable pressure, specific volume, and temperature (pvT) data by investigating alternative material properties, such as melt flow rate [63]. In addition, Hertz et al. introduced a dual-pump-drive machine that aids a smooth transition from the velocity-controlled to pressure-controlled phases, and improved operational consistency [64]. An alternative approach is presented by Lin et al., who developed a proportional-integral-derivative (PID) controller for screw retraction at the end of filling to allow for the necessary pressure drop [65].

**Focusing on the non-return valve (NRV):** It is imperative that switchover methods directly or indirectly take the closing behavior of the NRV into account. Still, the accurate detection of the closing time is a technically challenging process that cannot be easily accomplished [66]. Therefore, alternative approaches must be considered [67]. Identification by pressure gradient [45,46,49,68] or by a pressure threshold [67], for example 100 bar [5], are mentioned as methods for this detection.

Schiffers devised a screw guidance system that uses a dynamic adjustment of the injection rate profile contingent on the NRV’s sealing behavior. The system identifies the closing of the NRV through a pressure increase in the screw antechamber, calculates the requisite adjustments using a reference, and modifies the injection profile accordingly to maintain consistent performance. The injection profile is divided into a high- and a low-injection-rate step. By shifting the position of the change in the injection rate, an analogous effect to a change in the switchover point is achieved [45]. Heinzler also emphasized the importance of maintaining a consistent shot volume by monitoring a reference mass pressure curve and controlling the screw velocity in real time, as well as making adaptive adjustments to the switchover point. From the point at which the NRV closes, defined as the point at which the injection pressure reaches 100 bar, the system maintains a constant injection volume. The necessary changes are also used in conjunction with pvT data for the purpose of adaptive control of the holding pressure phase [5].

Eben outlines a switchover methodology that refers to the closure of the NRV instead of the start of the filling phase, using changes in the injection pressure as an indicator of NRV closure. This method eliminates the need for supplementary sensors and provides a more precise timing for the switchover by focusing on the moment of the uninterrupted melt flow following NRV closure [49].

To further enhance NRV functionality, Sumitomo (SHI) Demag’s activeLock system incorporates a lockable NRV that closes after metering by counter-rotating the screw, ensuring a reproducible closing process [69,70]. Furthermore, Engel Austria GmbH’s Smartshut system addresses variability in NRV behavior by employing a forced closing mechanism which serves to minimize the effects of inconsistent closing behavior [71,72,73]. Xaloy offers NRVs under the name Auto-Shut™ Valves that close independently of the screw travel. These are designed for PC and ABS alloys, as well as acrylic and polyolefins of 10 MI or less, but they are not suitable for nylons and glass-filled materials [74]. In addition, Fanuc has patented a free-rotating screw that reduces the forces from the screw flights opposing the closing of the NRV, thus speeding up the closing of the NRV [75].

Ma et al. developed a method to detect wear in NRVs, distinguishing between axial and radial wear. Axial wear, which causes slight delays in pressure buildup, can be compensated for by adjusting the process parameters. However, radial wear, which leads to significant pressure loss, requires the replacement of the NRV. An algorithm developed in this study provides online compensation for axial wear, ensuring consistent part quality [76]. Lastly, with regard to plasticizing, Liu proposed a concept in which the rotational speed for metering and backpressure are not regulated independently, thereby stabilizing the dissipative energy input. This approach has been demonstrated to reduce process fluctuations and helps minimize temperature variations, which directly affect the closing behavior of the NRV [77].

**Refining switchover methods for multi-cavities:** In a recent study, Párizs et al. investigated a pressure-dependent switchover for multi-cavities, employing pressure sensors within the cavity and in the runner system. The selection of a sensor at the end of the flow path is susceptible to fluctuations. A correspondingly elevated pressure increase in the runner system facilitated a dependable switchover. The pressure must be selected in such a way as to avoid the pressure increase due to the different cross-sections at the gates and to detect the pressure increase due to the compression after volumetric filling [78].

Furthermore, in the context of multi-cavities, particularly in the case of family molds with cavities of varying dimensions, the volumetric filling process can be a significant challenge. Without adequate precision and control, there is a risk of accumulating excessive pressure, which could lead to undesirable outcomes such as overmolding and the formation of burrs. ActiveFlowBalance, a solution offered by Sumitomo (SHI) Demag Plastics Machinery GmbH, can improve the efficiency of filling in such scenarios. By keeping the screw in the switchover position, the injection pressure can be released in the direction of the mold, preventing the overfilling of smaller cavities during filling and allowing the melt to flow into those that remain underfilled [22,25].

**Rethinking the pressure-dependent switchover:** The pressure-dependent switchover is triggered when a predefined threshold pressure value is exceeded. This should theoretically occur at the onset of compression following volumetric filling. However, due to external factors such as the viscosity of the substance in question, the actual threshold value may vary. To detect the compression onset with greater accuracy, Bader et al. used fuzzy logic to predict the switchover point based on the last three cavity pressure measurements [79]. Similarly, Gao and Zhao applied fuzzy logic to define the switchover point using pre-filtered nozzle pressure [80]. Since fuzzy inference systems depend on having a starting point and improving from there, these systems fail if the machine is not set up properly. Accordingly, Zhou et al. used case-based reasoning to identify a comparable process and transfer the parameter settings that serve as the foundation for fuzzy inference to optimize the process based on observed defects [81]. Huang implemented a gray model to predict cavity filling based on cavity pressure and a trigger switchover [38,82]. This method was then redefined with the addition of stroke position as another input [83]. Nian et al. established a decision rule to optimize parameter settings, including the timing of the switchover, through the observation of the stroke position and cavity pressure history profiles. As the cavity pressure increases rapidly, the corresponding stroke position is defined as the switchover point [24]. Other studies use the change in the gradient of the pressure curve instead of the pressure value alone. The patent from Scheckenbach et al. and the study of Tseng et al. both use a threshold based on the second derivative of the pressure curve as a criterion for determining the switchover point [84,85]. Gornik proposed an alternative methodology for a pressure-gradient-dependent switchover that does not rely on pressure over time but rather on pressure over volume. This approach offers a versatile solution that can accommodate diverse melt temperatures and injection rates [48].

### 4.2. Adaptive Process Control for the Switchover Point

Conventional switchover strategies in injection molding typically rely on fixed thresholds, making them sensitive to disturbances and process drift. In contrast, adaptive process control aims to dynamically adjust the switchover conditions based on previous cycle data or real-time sensor feedback, thereby improving robustness, part quality, and energy efficiency.

The early signs of adaptivity can be found in modified conventional approaches. For example, using the pressure gradient instead of a fixed pressure threshold for a switchover inherently accounts for viscosity-related variations. Similarly, plotting the cavity pressure versus the screw position instead of time helps compensate for changes in the injection rate, improving the robustness of the decision logic. These concepts are also embedded in several advanced strategies discussed in the following subsections, such as the use of pressure integrals. Furthermore, conventional stroke- or time-based switchovers can be refined by altering the reference signal. Instead of using the beginning of the filling phase, signals reflecting the closing of the NRV [49] or those from in-mold sensors that do not necessarily have to be placed at the end of the flow [4,44] provide more robust and physically meaningful reference points.

This section reviews recent contributions toward adaptive switchover control, including step-by-step optimization approaches, the use of pressure integrals as robust control signals, and the incorporation of deformation-based methods (mold separation, tie-bar elongation). In addition, emerging solutions based on real-time simulations and artificial intelligence are discussed as promising tools for future process automation.

**Step-by-step optimization:** Liou et al. proposed an optimization strategy that employs a sequential adjustment of four fundamental parameters: the switchover point, injection rate, holding pressure, and clamping force. To determine the optimal switchover point, this method uses pressure peaks during injection to identify the most suitable process settings, with the lowest pressure peak serving as the determining criterion. Liou’s approach can be utilized as a setup strategy and can also be applied to adaptive process control [10]. Liou’s optimization strategy, as well as the strategy as used by Cheng et al. [86], identifies a range of process parameters that should be targeted, with adjustments made incrementally to identify the optimal setup. To illustrate, if the pressure peak is excessive, the position-based switchover point is increased by 0.1 mm, and conversely, it is diminished by 0.1 mm [10,86]. Cho et al. have shown the effect of a switchover on the cavity pressure curve in their investigations. Based on this, the process could iteratively adjust the switchover point according to the desired cavity pressure curve. It is proposed that parameter optimization can be applied to adaptive control [23]. Similarly, Kumar et al. adapt the process using if–then queries. From the initial series of tests, data on the peak pressure, packing pressure, packing time, average pressure, peak temperature, and average temperature are extracted from the temperature and pressure profiles. These extracted values are then compared to their corresponding thresholds in order to detect any failure cases and adjust the process accordingly [87].

Although this step-by-step approach is effective, it can be time-consuming. In order to reduce the need for such gradual adjustments, many systems rely more heavily on reference data. By utilizing reference pressure curves from an ideal process cycle, systems can make real-time adjustments based on multiple process characteristics, enabling a more direct and automated response to fluctuations. Engel Austria GmbH employs this methodology using reference pressure curves which facilitate the continuous monitoring and adjustment of key parameters, such as the switchover point and injection rate [88]. Three principal categories of deviation in the pressure curve, changes in time/position, changes in slope, and unidentified deviations give rise to critical process indicators such as the shot volume, viscosity changes, and pressure curve uniformity. Subsequently, these indicators are used to improve the process and attain the desired curve profile [89,90].

**Pressure integrals:** The manner in which the NRV reaches its closing position may directly affect the shot volume. To maintain a constant specific injection work, which is represented by the pressure integral during injection, a method was patented by Sasaki et al. [91]. Kelly et al. propose the use of not only the complete pressure integral of the injection phase, which is indicative of the process, but also a second pressure integral that is representative of the material. The second integral is set in a range with minimal noise to exclude initial effects, such as the closing of the NRV. It corresponds to changes in viscosity and is therefore termed the viscosity index (*VI*). However, it did not show a strong correlation with the part weight due to insufficient interaction with other factors [92].

Similarly, the pressure integral over the complete filling stage was termed the filling index (*FI*) and is presented as the integrated pressure from the moment the NRV closes (e.g., identified by the pressure gradient [46,68] or by a pressure threshold [67]) to the switchover point. The conceptual differences between the *VI* and the *FI* are illustrated in Figure 3, which shows the corresponding pressure integrals for both approaches.

To establish a correlation between the pressure integrals and the part weight, the molded part volume equivalent (*MPV*) was introduced as the quotient of the *FI* and *VI*. It was found that keeping the *MPV* constant by adjusting the switchover point and other parameters results in a constant part weight [46,67,68,93]. This approach is shown by its implementation in KraussMaffei’s APC plus module [94]. Furthermore, the *VI* is also modified to some extent and is no longer calculated as a direct pressure integral. The pressure integral is multiplied by a correction factor, which is used to account for material compressibility and other factors. This is then divided by the average injection rate, thus obtaining the *VI* as described by [46,56,93,95]. Building upon this, Schiffers et al. present a method for determining the actual injection volume considering the material-specific compression. This allows the adjustment of the switchover point according to the defined filling volume, ensuring the continuous injection of the same volume. One reference cycle is needed to gather the desired thresholds in volume and the corresponding pressures. The required material-specific compressibility can be set manually or stored in the machine control as an adiabat compressibility curve, which would further improve the calculations [95].

Gao et al. used the *FI* and Su et al. used the *VI* to dynamically adjust the switchover point based on a pre-determined *FI* and *VI*, respectively [11,96]. 

Zhou et al. developed the pressure integral method for predicting the part weight and correspondingly adjusting the holding pressure [97]. Chen et al. adapted the *VI* to fine-tune the switchover point based on the specific properties of the material in question [98]. Fan-Jiang et al. and Xu et al. employed analogous methodologies to regulate the process, with Xu et al. emphasizing the maintenance of volume consistency through adjustments based on pressure and screw displacement data [12,99]. In their study, Liou et al. used an upper and lower bound for nozzle pressure, in conjunction with the *VI*, to enable the adaptive control of the injection rate and switchover point [9].

**Mold deformation, mold separation, and tie-bar elongation for switchover detection:** The mold-opening force applied to the mold can be quantified through a number of different methods, including the measurement of the clamping force, tie-bar elongation, mold deformation, or mold separation. These measurements are correlated with in-mold cavity pressure profiles. Smud et al. introduced a cross-phase approach to control cavity pressure during the filling and holding phases, including a variation in the control using mold separation instead of an in-mold sensor to adapt the holding phase, aiming to improve repeatability and part quality with both controls [100]. Similarly, Moritzer et al. developed an external cavity pressure measurement method using tie-bar elongation, which is particularly useful in cases like microinjection molding where internal pressure measurement is impractical. While fully hydraulic machines require calibration with reference curves due to limited responsiveness, electro-mechanical toggle machines have been shown to produce pressure profiles comparable to cavity pressure via tie-bar elongation [101].

In their 2006 study, Chen et al. investigated the use of mold separation as a control variable in an adaptive process control system. The initial stage of the process involves determining the settings through a simulation, which are then tested on the actual machine. Following this, the maximum mold separation and the hydraulic pressure at the switchover point are stored as references, having been tested and possibly adjusted. At the beginning of the control process, the switchover point is adjusted to set the maximum mold separation. Once the injection phase is properly regulated, the holding pressure control becomes active, adjusting the holding pressure to ensure that the mold separation follows the reference profile [102]. Building upon the findings, Chen and Turng employed mold separation in conjunction with other process variables (e.g., temperature, pressure, and stroke position) to refine the switchover point and to guarantee consistent mold separation, facilitating improved cycle-to-cycle control [103].

Zhang et al. investigated the relationship between mold separation and part weight, establishing a linear correlation. They introduced a mold-separation-based switchover method and compared it to traditional time-dependent switchover techniques. They found that the mold separation approach provided greater repeatability in component weight [104]. In a related contribution, Jaeger presented a comparable switchover method based on the deformation of the mold. In this study, an all-electric injection molding machine was used, and the deformation of the machine provided a signal that was identical to that of the cavity pressure. This enabled the reliable detection of the moment of volumetric filling, facilitating process-dependent switchover control that could adapt to external disturbances. However, Jaeger observed that this method is not transferable to hydraulic machines due to the lack of significant deformation in the clamping unit, suggesting instead the use of screw deceleration as a switchover signal for such machines [105].

Tie-bar elongation represents another approach to switchover detection. In their 2019 study, Zhang et al. used ultrasonic sensors on tie-bars to indirectly measure mold cavity pressure, using this data to adjust the switchover point and to reduce pressure peaks [14]. Chen et al. use the difference between the measured clamping force and a reference to increase or decrease the switchover time by one increment, optimizing part weight stability [106]. Building on these findings, Chen et al. and Huang et al. introduced a method that used tie-bar elongation profiles to determine the switchover point, offering a cost-effective alternative to traditional cavity pressure sensors. This approach aimed to reduce defects by ensuring precise control over the filling-to-packing switchover timing [16,17]. Subsequent studies by Chen et al. continued to explore the use of tie-bar elongation, with methods that adjusted the switchover point and holding pressure based on strain gauge measurements, showing significant improvements in part quality stability and the yield rate [1,2].

**Implementing the filling simulation:** The simulation of filling processes has received increased attention. Arburg GmbH in collaboration with Simcon and Engel Austria GmbH in collaboration with Autodesk and Simcon have devised a method for transferring data from the simulation stage to the injection molding machine. These simulations facilitate the visualization and prediction of mold filling, rendering adjustments more transparent and manageable [107,108,109]. Duffner and Filz also identified limitations in simulation precision for more complex molds, particularly where idealized models fail to account for variables like large hot runner systems [110]. However, due to the complexities of the switchover process and the challenges previously discussed, such as accurately determining the switchover point, simulations can only provide a reference value rather than a definitive solution. To address this problem, Johnston et al. introduced an online simulation using real-time nozzle pressure data. This simulation was used to predict the position of the melt front and trigger the switchover point. Although this method has proven to be effective, it was susceptible to increased process variance due to time delays in data transmission and adaptation. To improve accuracy, improvements in computing power and the inclusion of additional process data, adaptive viscosity models, and melt compressibility were suggested as potential avenues for further investigation [111]. 

**Utilizing artificial intelligence:** Huang et al. improved machine performance by employing closed-loop neural networks to regulate the injection rate and nozzle pressure, although the switchover point remained fixed [112]. Zhao et al. investigated the potential of predictive models, including Gaussian Processes and neural networks, to estimate the mold cavity pressure. Their findings suggest that these models may offer a viable alternative to traditional sensors, though they require additional inputs, such as the mold temperature, to achieve optimal accuracy [15,113]. Zhou et al. employed a sparse autoencoder to extract features from the injection pressure curve, enabling dynamic adjustments to the switchover point based on changes in the melt front rate [114]. Similarly, Guo et al. incorporated the switchover point as an input in a neural network model for predicting warpage, ensuring that its effect was considered during the optimization process [115]. Huang et al. employed an autoencoder and a multilayer perceptron with the objective of achieving a more precise alignment between the simulated and actual conditions [116]. Tsai et al. used a back-propagation neural network to iteratively adjust the switchover point and injection rate, maintaining process variables within narrow tolerances [117]. Wu et al. identified key factors, including the switchover position and clamping force, as being particularly important regarding cavity pressure. They proposed the implementation of an adaptive control system capable of adjusting these parameters in real time [118]. Borchardt et al. used a Bayesian network to predict the part weight. As Bayesian networks support both forward and backward inference, they can also be used to determine the most likely input parameters required to achieve a desired part weight [119].

### 4.3. Summary of Findings

The findings of this review, summarized in Table 1, explore switchover techniques and adaptive control methods for optimizing the switchover point. These findings are grouped into clusters of similar approaches and categorized by the type of implementation: “shot-to-shot” methods, applicable to subsequent cycles, and “in-cycle” methods, operating within the same cycle.

Advances in switchover methods emphasize improved robustness and adaptability. For example, pressure change detection has emerged as a more robust alternative to conventional pressure thresholds. This technique detects pressure increases through gradient changes, mitigating the effects of viscosity variations. However, it can be affected by injection rates. Gornik [48] suggests calculating the pressure gradient over volume rather than time, which provides a more consistent metric and reduces the effect of injection rate variations. 

To better quantify process stability and consistency in terms of pressure behavior during injection molding, several key indices based on pressure curves can be used.

**Figure 4:**
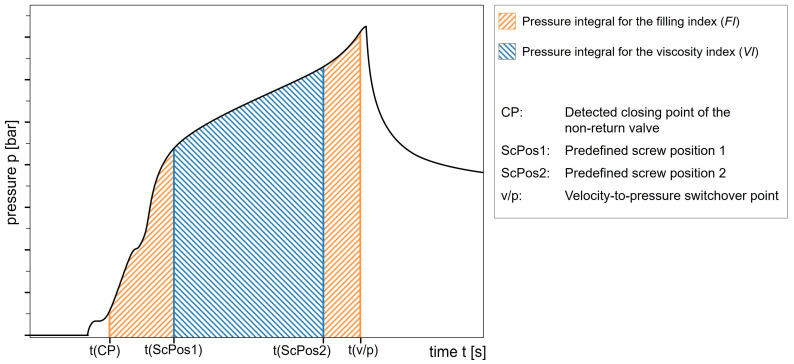
Representation of the pressure integrals for the viscosity index (*VI*) and filling index (*FI*). Own illustration based on the schematic in Figure 1 of [67].

The viscosity index (*VI*), as previously illustrated in Figure 4, represents the pressure integral for predefined screw positions or times, excluding influences from the startup and switchover [98]:

(1) $$VI=\int_{t\left(ScrewPosition1\right)}^{t\left(ScrewPosition2\right)}p\left(t\right)dt$$

A corrected version of the *VI* accounts for variations in injection rate v and the melt compressibility $c_{1}$ [56]:

(2) $${VI}_{corrected}=\int_{t\left(ScrewPosition1\right)}^{t\left(ScrewPosition2\right)}p\left(t\right)dt\times \frac{c_{1}}{v}$$

The filling index (*FI*), illustrated in Figure 4, represents the pressure integral from the closing of the non-return valve (NRV) to the switchover point [56]:

(3) $$FI=\int_{t(Closing\;of\;NRV)}^{t(Switchover)}p(t)dt$$

The molded part volume equivalent (*MPV*) is defined as the ratio of the *FI* to the *VI* [56]:

(4) $$MPV=\frac{FI}{VI}$$

Adaptive control methods allow dynamic adjustments to match the reference cycle criteria. This approach has also helped optimize stroke-dependent and time-dependent switchover by adaptively refining the reference start points. 

Instead of in-mold sensors, novel switchover strategies incorporate the detection of the mold-opening force associated with cavity pressure using methods such as the clamp force measurement, mold separation, or tie-bar elongation.

Furthermore, machine learning techniques facilitate the mapping of relationships between process parameters and system features, enabling real-time switchover adjustments. Complementary feature extraction methods assist in predicting proper switchover points, while ultrasonic sensors improve cavity pressure predictions. These ultrasonic sensors can also detect the melt front without placing a sensor in the cavity, eliminating potential mold damage. Similarly, updated real-time simulations based on nozzle pressure can be used to achieve the same objective.

While each approach offers specific benefits depending on the application, the findings indicate that no particular switchover method consistently outperforms the others. The selection of the most suitable strategy depends on the process conditions, expected viscosity changes, rheological and pvT properties of the material, quality criteria, and mold geometry such as wall thickness. The implementation of adaptive process control strategies and methods that employ detection of the melt front inside the cavity can help eliminate numerous influencing factors and provide more robust and consistent switchover control.

## 5. Perspectives for Future Research

The repeatability of the switchover has been studied in many ways, with the aim to improve it. These studies mainly use methods that exclude and thus minimize influencing factors. The detection of the melt front and its use for a switchover is less dependent on other process conditions, such as NRV closure. To avoid the problems of the in-mold sensor, numerous tasks for detecting the state of the melt in the cavity have been carried out using external sensors. Therefore, the detection of the melt front inside the mold via sensors offers further potential. Müller et al. analyzed the melt front detection through a mechanical actuator and verified the melt front detection through a cavity temperature sensor, while also proposing the usage of the ejector pins [121].

Additionally, switchover methods could enable adaptive adjustments in response to process changes. For this application, investigations are necessary for all types of switchover methods, including those based on the detection of changes in pressure, to better understand the impact of factors, like the injection rate, and to ensure their robustness under varying process conditions. A particular focus on analyzing the consistency and reliability of switchover methods based on pressure change detection over stroke position, rather than time, could help mitigate the effects of process variations, such as NRV closing behavior, and process adaptations, such as the injection rate.

Further investigation into the transferability of these results to other materials and part geometries appears to be necessary. In this context, for deformation-based methods (mold separation, tie-bar elongation), additional studies on the required projected area and the influence of wall thickness are of great interest to improve their applicability and accuracy.

With the advances in the area of digitalization, digital twins are an attractive area of research, with obvious benefits for the active control of the process and the switchover point. Updating and adapting the simulation with actual process data is crucial, offering potential areas for further research. Determining the compressibility or additional pressure, specific volume, and temperature (pvT) data within the process would also improve the calculations of the injected volume or more comprehensive simulations. Methods for in-line determination of melt compressibility are suggested by Gornik [122] and Pillwein [123].

Machine learning approaches also offer further potential. As the switchover point depends on the current conditions, the resulting process variables should be used as a basis. The process curves for early, late, and proper switchovers differ, so this can be used for classification or feature extraction. For a transferable approach, the resulting process variables, such as the injection pressure over time, should be used as training data. A classification approach using such data has been presented by Rottenwalter et al. [124].

Most of the literature relates to thermoplastic injection molding, so the transferability of the methods to other injection molding processes provides an area for further investigation. For the thermoset injection molding of bulk molding compounds (BMCs), NRVs are used, so the adaptive adjustments of the switchover position, which compensate for the closing behavior of the NRV, are applicable here [125]. Liquid silicone rubber (LSR) injection molding uses a screw system designed to mix and convey the LSR and not compress it, so a special NRV consisting of a bushing seat and spring is used [126]. Barbaroux et al. [127] determined an influence of the switchover point on the part weight, but only for hot runner systems and not for cold runner systems. For these LSR injection molding processes, methods which detect the closure of an NRV may help for compensation. Polymer injection forming is used to produce sheet metal/polymer macro-composite parts in a single manufacturing step. Baesso et al. [51] have shown that the weight fluctuations of traditional injection molding, as well as polymer injection molding, were reduced by the use of a cavity pressure switchover compared to a stroke- and pressure-dependent switchover. Microinjection molding machines have either a barrel and screw with reduced dimensions or a separate metering unit, which allows for an injection unit with a smaller plunger, improving positional control accuracy [128,129]. A stroke dependent switchover is recommended for microinjection molding [128]. External cavity pressure measurement methods, such as tie-bar elongation as recommended by Moritzer et al. [101], may be applicable since in-mold sensors are impractical for microinjection molding. Further research regarding the transferability of additional switchover approaches is recommended.

## 6. Conclusions

The findings indicate that no particular switchover method consistently outperforms the others. The selection of the ideal switchover method is mainly dependent on the injection process, the expected viscosity changes, the rheological and pvT properties of the material, the quality criteria, and the mold geometry such as the number of cavities and the wall thickness. Adaptive process control and methods that use sensors that detect the melt at the end of the flow path can eliminate many of the influencing factors, allowing for a more robust switchover.

Research on switchover methods has focused on reducing influencing factors affecting the switchover point and the adaptive control of the switchover point to improve robustness for processing and material variations. Smoothening the transition between the filling and holding phases has been identified as a potential area for enhancement. Additionally, compensating for variations in the closing behavior of the NRV has been shown to improve switchover robustness. 

While the integration of adaptive control modules is a feature offered by some machine manufacturers, certain developments in this area hold promise for broader integration into machine control systems. For instance, determining the switchover point based on pressure changes along the screw position, rather than using fixed pressure thresholds, has demonstrated improved robustness and adaptability compared to conventional pressure-dependent strategies. The incorporation of these methodologies into standard control logic has the potential to facilitate more stable and reproducible processing conditions, particularly in the context of material or process fluctuations.

## Figures and Tables

**Table 1:** Novel switchover methods or adaptations. The superscripts “patent” mark indicate which patents are referenced. The superscripts “proposed” are used to indicate references that propose a switchover method or adaptation, without providing experimental research to validate the proposition.

Cluster	Shot-to-Shot	In-Cycle	Mold Geometry	Material	Machine	Quality Criterion
**Pressure-gradient-dependent switchover**	Extracting the correspondent screw position for a sudden pressure raise [24,85]	Detecting sudden pressure rise: Threshold in second derivative [84]^patent^ Fuzzy logic [79,80]^patent^ Case-based reasoning and fuzzy logic [81] Gray model [38] [82,83]^patent^ Using pressure over volume instead of time [48]^proposed^	**Flat** [1,2,4,5,9,10,11,12,14,15,16,17,38,45,49,76,78,89,93,99,102,103,104,105,106,111,113,115,117,118,120] **Box-shaped** [24,81,85,86,93,98,114,119] **Complexity above box-shaped** [56,68] **N/A** [13,31,46,101]	**PP** [4,9,10,11,12,14,49,76,86,89,93,98,99,102,103,104,111,115,117,119] **HDPE** [45] **LDPE** [14] **PA6** [5,45,93] **PA66 GF30** [56] **PBT** [5,45] **PS** [38,105,113,118] **PPE GF30** [24] **PPA** [46,68] **ABS** [1,2,16,17,78,81,106,119] **PC** [38,56,68,102,103,120] **PC-ABS** [56,68] **PVC** [15,113] **SAN** [85] **N/A** [13,31,101,114]	**Electric** [1,2,5,14,16,17,45,46,49,68,76,86,89,93,99,101,105,106,111,119] **Hydraulic** [4,9,10,11,12,24,38,56,68,78,85,98,99,101,102,103,113,117,118] **N/A** [13,31,81,104,114,115,120]	**Weight** [1,2,4,5,9,10,11,12,14,17,38,45,46,49,56,68,76,78,86,89,93,98,99,102,103,104,105,106,111,113,118,119,120] **Dimensions** [4,49] **Warpage** [24,115] **Peak pressure, peak pressure time, and viscosity index** [117] **Surface characteristics** [5] **Simulation image** [114] **Correlation with cavity pressure curves** [15,16,101] **Ultrasonic signal** [13] **Filling stage** [78,81,85] **Flash** [78]
**Utilizing reference data**	Adjusting the switchover point to keep parameter constant: Nozzle peak pressure [10,86] Maximum clamping force [106] Constant mold separation [102,103] Tie-bar elongation [16]^proposed^ [1,2,17] Pressure integral (viscosity index) [11,98] Peak pressure and pressure integral (viscosity index) [9,12]	Trigger switchover point according to the following: Mold separation [104] Clamping plate deformation [105]^proposed^ Tie-bar elongation [14,101]^proposed^ Pressure integral: filling index [91,96]^patent^ Pressure integral: Viscosity Index [99] Pressure integral: molded part volume equivalent [46,56,68,93] [67]^patent^ Adjustments, according to alterations in pressure curve characteristics such as time/position, changes in slope, and unidentified deviations [89] [88,90]^patent^				
**Adjusting the reference for time- or stroke-dependent switchover**	Closure of NRV: Delayed pressure buildup [76]	In-mold sensor [4,120] Closure of non-return valve detected through the following: Pressure gradient [45,49] Pressure threshold [5]				
**Machine learning**	Predicting a switchover point through the following: Feature extraction [114] Adjust the switchover point to optimize quality criterion: Pretrained machine learning model [115,117,118,119] Genetic algorithm [115]	Predicting cavity pressure with the following: Gaussian process (inputs: melt density, calculated based on the ultrasonic reflection coefficient, along with mold temperature and hydraulic pressure) [15]^proposed^ Feed-forward neural network [15,113]^proposed^				
**Other switchover methods**		Screw deacceleration [31] [105]^proposed^ Pressure sensor in the runner system for multi-cavities [78] Melt front detection without in-mold sensors: Simulation-based [111] Ultrasonic sensor (external) [13] Feed-forward neural network (ultrasonic sensor, mold temperature, hydraulic pressure) [15,113]^proposed^				

## Data Availability

No new data were created or analyzed in this study. Data sharing is not applicable to this article.

## Abbreviations

The following abbreviations are used in this manuscript:

NRV	Non-return valve
VI	Viscosity index
FI	Filling index
MPV	Molded part volume equivalent
pvT	Pressure, specific volume, and temperature
